# Vitamin D: Nutritional Programming During the First 1000 Days of Life

**DOI:** 10.3390/nu18071096

**Published:** 2026-03-29

**Authors:** Costanza Sortino, Maurizio Carta, Cristina Bonacasa, Eva Candela, Veronica Notarbartolo, Laura Maria Sollena, Mario Giuffrè

**Affiliations:** 1 Neonatal Intensive Care Unit, Department of Health Promotion, Mother and Child Care, Internal Medicine and Medical Specialties “G. D’Alessandro”, University of Palermo, 90127 Palermo, Italy; costanza.sortino@community.unipa.it (C.S.); lauramaria.sollena@community.unipa.it (L.M.S.); mario.giuffre@policlinico.pa.it (M.G.); 2 Neonatology and Neonatal Intensive Care Unit, University Hospital Policlinico “Paolo Giaccone”, 90127 Palermo, Italy; cristina.bonacasa@policlinico.pa.it (C.B.); eva.candela@policlinico.pa.it (E.C.)

**Keywords:** vitamin D, first 1000 days, nutritional programming, pregnancy, infancy, early childhood, epigenetics, growth

## Abstract

Background: The first 1000 days of life represent a critical window for developmental programming, during which specific nutritional exposures, such as vitamin D levels, may influence long-term health trajectories. Vitamin D plays a central role in skeletal development, but increasing evidence also supports its possible involvement in immune, metabolic, and neurodevelopmental processes during early life. In this narrative review, we summarize current evidence on the biological functions of vitamin D across the first 1000 days, focusing on its roles in skeletal, immune, metabolic, and neurodevelopmental processes, and its potential role as a programming factor. Methods: We conducted our research using the PubMed, Scopus, and Cochrane databases. We included systematic reviews, randomized controlled trials, and high-quality observational studies published from 2015 onward, focusing on pregnancy, neonatal life, and early childhood. Results: Vitamin D acts through placental, epigenetic, skeletal, immune, metabolic, and neurodevelopmental pathways that are particularly active during early development. Low maternal or early-life vitamin D status has been associated with adverse birth outcomes and impaired bone health. It has also been linked to increased susceptibility to infections and allergic diseases, altered metabolic trajectories, and mild neurodevelopmental differences. Evidence from supplementation trials remains heterogeneous, with benefits appearing more consistent in populations with baseline deficiency. Conclusions: Vitamin D fulfills several biological plausibility criteria for a potential early-life programming factor, although current human evidence remains heterogeneous.

## 1. Introduction

**Figure 1:**
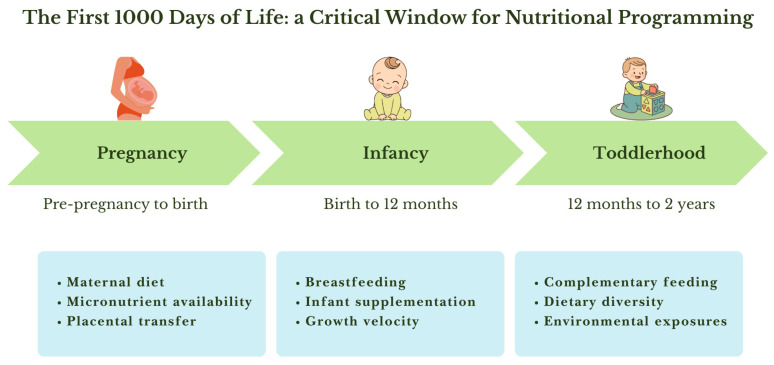
Key maternal, infant, and environmental factors influencing nutritional programming during pregnancy, infancy and toddlerhood.

The first 1000 days of life represent a critical developmental window characterized by rapid growth and increased sensitivity to a variety of environmental factors. Environmental stimuli during this period shape early health outcomes and may also trigger long-lasting biological adaptations that influence disease risk later in life [1]. During this time frame, which comprises intrauterine life, the neonatal period, and the first two years of life, certain factors may have a positive effect, representing a “window of opportunity”, while others may increase the risk of disease development, constituting a “window of susceptibility” (Figure 1) [2]. Among these environmental influences, early-life nutrition represents a key and modifiable factor, playing a central role in fetal programming in utero, in postnatal growth and development, and in the establishment of metabolic health in adult life [1]. Insufficient intake of essential nutrients during the first 1000 days of life may result in developmental limitations, potentially causing enduring effects on brain function. For this reason, particular attention to the adequacy of essential nutrient intake is required to support optimal development and promote maternal health during pregnancy [3]. In the context of early-life nutrition, vitamin D has gained particular attention during the first 1000 days of life because of its relevance to maternal health, fetal development, and early postnatal growth. In fact, vitamin D plays a key role in regulating calcium and phosphorus homeostasis, as well as in bone mineralization and bone mass acquisition. In recent years, however, interest in vitamin D has extended beyond skeletal functions, as emerging evidence indicates that vitamin D also exerts important extraskeletal effects, potentially contributing to the pathogenesis of several conditions, including infectious and autoimmune diseases [4]. Because neonatal 25(OH)D levels depend almost entirely on maternal stores, infants of mothers with vitamin D deficiency face a heightened risk of hypocalcemia, rickets, and infectious illnesses throughout early life. Moreover, a cross-sectional observational study analyzing umbilical cord blood samples at birth reported a high prevalence of vitamin D deficiency in pregnant women, highlighting the close dependence of neonatal vitamin D status on maternal concentrations [5]. In this context, understanding the role of vitamin D during the first 1000 days is particularly relevant, as this period represents a critical window in which nutritional exposures may shape lifelong health trajectories. Although numerous studies have examined vitamin D status in pregnancy and early infancy [6], the evidence regarding its potential contribution to developmental programming remains fragmented and, in some areas, controversial, especially with respect to immune and metabolic outcomes [7]. Within the developmental origins of health and disease (DOHaD) framework, nutritional programming refers to the process by which environmental exposures during critical windows of development induce persistent structural, functional, or regulatory changes that influence long-term health trajectories. For a nutrient to plausibly act as a programming factor, several criteria should be met, including biological plausibility, temporally restricted sensitivity, potential for lasting effects beyond the exposure period, and consistency across mechanistic and epidemiological evidence [8,9,10]. Therefore, the present review aims to synthesize current knowledge on the role of vitamin D across the first 1000 days, evaluate the strength of available evidence, and identify gaps in the literature. By integrating findings from observational studies, randomized trials, and mechanistic research, this review examines the evidence supporting vitamin D as a potential contributor to developmental programming during the first 1000 days of life, while critically assessing the strength and limitations of the available data.

## 2. Methods

A narrative review was performed according to the most recent available literature. Although no date restrictions were applied in the search strategy, this review prioritizes evidence published within the past decade (from 2015 onward) to ensure the inclusion of the most up-to-date and clinically relevant data. The research was conducted using the following keywords (alone or in combination): vitamin D, first 1000 days, pregnancy, infancy, early childhood, nutritional programming, epigenetics, growth, immune development, metabolic outcomes and supplementation. PubMed, Scopus and Cochrane were used as the electronic databases. Study selection was guided by relevance to the research question, methodological quality, and contribution to understanding biological plausibility or clinical outcomes. Systematic reviews, meta-analyses, randomized controlled trials, and high-quality observational studies were preferentially included. Mechanistic studies were considered when they provided insights into potential programming pathways. As this work was designed as a narrative review, no formal quality scoring system was applied; on the contrary, studies were discussed based on their methodological characteristics, relevance to the research question, and the overall consistency of the available evidence.

## 3. Vitamin D: Sources, Metabolism and Physiological Activation

The term vitamin D refers to two distinct compounds present in nature: vitamin D_3_ (cholecalciferol), originating from animal tissues, and vitamin D_2_ (ergocalciferol), produced by plants and fungi. Vitamin D_3_ is synthesized in human skin upon exposure to sunlight, while both D_2_ and D_3_ can also be acquired through dietary intake [4,11]. Upon UVB exposure, 7-dehydrocholesterol in the skin is converted into previtamin D_3_, which then undergoes thermal isomerization to form vitamin D_3_. Cutaneous synthesis is tightly regulated: excess UVB leads to the formation of inactive photoproducts, preventing vitamin D intoxication [11,12]. Both vitamin D_2_ and Vitamin D_3_ can also be obtained from dietary sources, including oily fish, eggs, UV-exposed mushrooms and fortified foods. Notably, a recent European analysis reported that fortified fats and spreads were the major contributors to total dietary vitamin D, followed by eggs, seafood, meat and poultry, and fortified dairy or plant-based milk products; moreover, animal-source foods—including meat, poultry, eggs, processed meats, and cheese—represented the main dietary sources of 25(OH)D_3_ [13]. Once formed, vitamin D_3_ is released into the circulation bound to vitamin D-binding protein (DBP), whereas dietary vitamin D_2_ and D_3_ are incorporated into chylomicrons and enter the bloodstream via the lymphatic system [14]. Both forms undergo 25-hydroxylation in the liver (via CYP2R1) to generate 25-hydroxyvitamin D [25(OH)D], the primary circulating metabolite used to assess vitamin D status [15]. In the kidney, 25(OH)D is further converted by CYP27B1 to the active hormone 1,25-dihydroxyvitamin D [1,25(OH)_2_D] [16]. This process is regulated by PTH, calcium, phosphate, FGF-23 and negative feedback by 1,25(OH)_2_D itself [17]. 1,25(OH)_2_D exerts its biologic functions primarily through the nuclear high-affinity vitamin D receptor (VDR) [18], whose tissue distribution and functional implications will be discussed in the following sections. 1,25(OH)_2_D plays a central role in calcium–phosphate homeostasis by enhancing active intestinal calcium absorption, regulating renal calcium reabsorption, and supporting normal growth plate function. These actions are essential for adequate skeletal mineralization, especially during periods of rapid growth such as fetal life and early childhood [18,19]. These well-established physiological mechanisms are summarized here to provide the biological framework necessary to understand how vitamin D exposure during the first 1000 days of life may influence developmental programming processes.

## 4. Vitamin D During the First 1000 Days: Biological Actions and Health Outcomes

The following sections summarize evidence on key physiological systems that may be influenced by vitamin D exposure during the first 1000 days of life, with particular attention to pathways potentially involved in developmental programming.

### 4.1. Skeletal Development and Bone Health Across the First 1000 Days

Skeletal development during the first 1000 days of life is characterized by rapid mineral accretion and high biological plasticity, making the developing skeleton particularly susceptible to nutritional and hormonal influences [20,21]. Vitamin D contributes to skeletal health primarily through its role in regulating calcium–phosphate homeostasis, which supports fetal and early postnatal bone mineralization [20,22]. During pregnancy, maternal adaptations—including increased intestinal calcium absorption and tightly regulated placental calcium transport—largely attenuate the effects of moderate variations in maternal vitamin D status on fetal skeletal mineralization [20]. As a result, while maternal and cord-blood 25-hydroxyvitamin D concentrations are closely correlated, the impact of prenatal vitamin D status on fetal bone development appears most relevant in conditions of marked deficiency or limited mineral availability, providing a biological framework for the heterogeneous skeletal outcomes reported in human studies [21,23]. In parallel, biomechanical forces play an important role: fetal bone loading has been identified as a key determinant of bone strength in fetal life and early infancy, suggesting that vitamin D-dependent mineral availability interacts with mechanical stimuli to shape early skeletal architecture [22]. Building on this biological framework, interventional evidence from randomized controlled trials aimed to clarify whether improving maternal vitamin D status during pregnancy translates into measurable benefits for offspring skeletal outcomes. The MAVIDOS trial, a multicenter, double-blind, placebo-controlled randomized study conducted in pregnant women in the United Kingdom, demonstrated that supplementation with cholecalciferol at a dose of 1000 IU/day from mid-pregnancy until delivery resulted in higher neonatal whole-body bone mineral content at birth, with effects most evident among infants born during winter months, highlighting the importance of baseline status, seasonal ultraviolet B exposure, and timing of intervention [24]. In a subsequent childhood follow-up analysis of the MAVIDOS cohort at 6–7 years of age, children exposed to prenatal vitamin D supplementation exhibited higher whole-body and lumbar spine bone mineral indices, providing evidence that gestational vitamin D exposure may influence skeletal accrual trajectories beyond the neonatal period, although the clinical relevance for peak bone mass remains to be established [25]. At the level of synthesized evidence, a recent systematic review and meta-analysis by Chien et al. examined randomized controlled trials of vitamin D supplementation conducted in pregnant women, with skeletal outcomes assessed in their offspring at birth or during early infancy. This analysis showed that gestational vitamin D supplementation consistently improves maternal and cord-blood 25-hydroxyvitamin D concentrations, whereas effects on offspring skeletal outcomes—including bone mineral content and related indices—were heterogeneous across studies and appeared to depend on baseline maternal vitamin D deficiency, calcium intake, supplementation dose (typically ranging from 400 to 4000 IU/day across trials), timing of intervention, and trial design [26]. Similarly, a comprehensive review by Moon et al. integrating evidence from both randomized and observational pregnancy studies reported variable associations between prenatal vitamin D status and neonatal skeletal measures, despite a strong and consistent correlation between maternal and cord-blood 25-hydroxyvitamin D concentrations [23].

Beyond subtle variations in bone mass, severe vitamin D deficiency during pregnancy and infancy is a well-established risk factor for nutritional rickets, a preventable disorder characterized by impaired mineralization of the growing skeleton [27]. The Global Consensus Recommendations identify severe vitamin D deficiency—often in combination with insufficient dietary calcium—as a primary etiological driver of defective mineralization of the growing skeleton in infants and young children, emphasizing prevention through adequate vitamin D supply during infancy and other high-risk windows [28]. Specific evidence in breastfed infants further highlights the clinical relevance of postnatal exposure: a systematic review of trials in term breastfed infants and mother–infant pairs by Tan et al. (19 studies; 2837 pairs) showed that direct infant supplementation improves infant 25-hydroxyvitamin D status, but that hard skeletal endpoints such as bone mineral content or rickets are infrequently captured in generally healthy term cohorts, limiting certainty for clinical outcomes despite consistent biochemical effects [29]. Alternative strategies have also been evaluated: O’Callaghan et al. reviewed maternal postpartum (“M-PP”) and infant intermittent (“I-INT”) regimens as potential approaches to achieve vitamin D sufficiency in breastfed infants, while emphasizing that comparative efficacy and especially comprehensive safety data remain insufficient to replace daily infant prophylaxis in routine practice [30]. Comparative dosing evidence in early life is further informed by a 2024 systematic review and Bayesian network meta-analysis by Abiramalatha et al., including term and late-preterm neonates who initiated enteral vitamin D supplementation within the first 6 weeks of life. Across twenty-nine trials and fourteen regimens, daily doses ≥400 IU consistently increased mean 25-hydroxyvitamin D concentrations compared with no supplementation, whereas lower daily doses and intermittent weekly regimens were ineffective, and higher daily doses (≥800 IU) were associated with an increased risk of hypervitaminosis D and hypercalcemia. Overall, these findings support daily supplementation around 400–600 IU as the most favorable balance between efficacy and safety in most settings [31]. Furthermore, evidence that maternal vitamin D supplementation can reduce infant rickets risk in high-risk settings is provided by a 2024 secondary analysis by Lautatzis et al. of the MDIG randomized, placebo-controlled dose-ranging trial conducted in Bangladesh. In this study, high-dose maternal cholecalciferol supplementation (28,000 IU/week) continued through 6 months postpartum was associated with a markedly lower prevalence of biochemical rickets at 6–12 months of age, whereas prenatal supplementation alone did not confer comparable protection [32]. Finally, implementation remains a key determinant of real-world effectiveness: using Canadian survey data, Weiler et al. reported that most breastfed infants received vitamin D supplementation, but daily adherence was not universal and was patterned by sociodemographic factors, underscoring that preventing vitamin D deficiency and rickets depends not only on recommendations but also on sustained uptake in routine care [33].

### 4.2. Immunomodulatory Actions and Immune-Related Outcomes

Vitamin D plays a key role in shaping both innate and adaptive immunity through the widespread expression of the vitamin D receptor (VDR) and vitamin D-dependent signaling pathways in innate and adaptive immune cells, including macrophages, dendritic cells, and T lymphocytes [34]. The vitamin D receptor is a ligand-dependent transcription factor expressed in most nucleated cells, through which vitamin D regulates the transcription of more than 1000 genes [35,36]. In addition to renal activation, several immune and epithelial tissues express the 1α-hydroxylase CYP27B1, enabling local conversion of 25(OH)D into its active form and supporting autocrine and paracrine responses [37]. Vitamin D influences both innate and adaptive pathways. Pathogen-related stimuli upregulate CYP27B1 in immune cells, promoting local synthesis of calcitriol, which enhances antimicrobial activity (e.g., cathelicidins and defensins), modulates NF-kB signaling, promotes regulatory T-cell differentiation, and reduces pro-inflammatory cytokines such as IL-6 and TNF-α while increasing IL-10 production [38]. Vitamin D also contributes to epithelial-barrier integrity and shapes mucosal defense, partly by modulating pathogen-recognition mechanisms and inflammasome activity, thereby supporting host protection at respiratory and intestinal surfaces [39,40]. These mechanisms are particularly relevant during the first 1000 days of life, a critical window for immune maturation and developmental programming, during which prenatal and early postnatal vitamin D exposure may influence the establishment of immune trajectories with potential long-term effects on host defense and immune tolerance.

Regarding infectious outcomes, clinical evidence supports a protective role of vitamin D supplementation against acute respiratory tract infections during early life, with effects that are strongly dependent on dosing regimen and baseline vitamin D status. In a large individual participant data meta-analysis of 25 randomized controlled trials including participants from birth to older adulthood, Martineau et al. showed that vitamin D supplementation was associated with a reduced risk of acute respiratory tract infections, with protective effects confined to daily or weekly dosing schedules, particularly at daily equivalent doses below 2000 IU, and not observed with intermittent high-dose bolus administration. The magnitude of benefit was greatest among individuals with low baseline serum 25-hydroxyvitamin D concentrations [41]. An updated aggregate data meta-analysis by Jolliffe et al., including more than 40 randomized controlled trials and over 48,000 participants, confirmed a small overall reduction in acute respiratory infection risk associated with vitamin D supplementation, while highlighting substantial heterogeneity across studies. In this analysis, protective effects were most consistently observed in trials using daily dosing regimens, at daily equivalent doses of 400–1000 IU, with a duration of 12 months or less, and among children aged 1–15 years, whereas no significant benefit was observed in infants younger than 1 year or with intermittent high-dose bolus administration [42]. Infant-specific evidence is provided by a large prospective birth cohort study conducted by Hong et al., which examined healthy, full-term infants followed from birth to six months of age. In this study, routine vitamin D supplementation started at birth, at daily doses of 400–600 IU, was associated with a lower incidence of respiratory tract infections, including lower respiratory tract infections and infection-related hospitalizations, during the first six months of life. Importantly, the protective association showed a frequency-dependent pattern, with the lowest infection risk observed among infants receiving supplementation on most days of the week, supporting the relevance of regular low-dose supplementation during early infancy [43].

Beyond infections, early-life vitamin D exposure has been investigated for its potential role in immune tolerance and the development of allergic disease. Direct clinical evidence comes from the New Zealand randomized, double-blind, placebo-controlled trial by Grant et al., conducted in pregnant women from 27 weeks gestation to delivery and their infants from birth to 6 months. Mother–infant pairs were randomized to placebo/placebo or to daily vitamin D at 1000 IU/day in pregnancy plus 400 IU/day in infancy, or 2000 IU/day in pregnancy plus 800 IU/day in infancy. At 18 months, the higher-dose regimen was associated with a lower proportion of children sensitized to house dust mite aeroallergens (specific IgE/skin testing), and there was also a signal for fewer primary-care visits in which asthma was diagnosed [44]. Evidence focused on asthma/wheeze prevention is further informed by VDAART. In this multicenter randomized, double-blind, placebo-controlled trial, pregnant women with a personal or family history of allergy/asthma were enrolled at 10–18 weeks’ gestation and assigned to 4000 IU/day vitamin D plus a prenatal multivitamin containing 400 IU/day (total 4400 IU/day) versus placebo plus the standard prenatal multivitamin (400 IU/day). Long-term follow-up analyses extending into later childhood and adolescence indicate that prenatal supplementation may influence asthma-related trajectories, with effects that appear sensitive to maternal baseline vitamin D status and other modifiers [45]. For eczema and food allergy, the overall picture is less consistent. A 2023 systematic review (including 6 randomized trials plus prospective observational studies) concluded that vitamin D supplementation during pregnancy showed no clear reduction in eczema incidence overall, and randomized evidence for food allergy outcomes was also largely null. In contrast, observational evidence suggested that higher cord-blood 25(OH)D may be associated with a lower risk of eczema around 1 year of age, highlighting heterogeneity by exposure window (maternal vs. cord blood vs. infancy) and outcome definition [46]. Finally, ongoing evidence generation should be acknowledged. A 2024 publication describes the design of two large double-blind, randomized controlled trials evaluating prenatal supplementation with vitamin D and fish oil, alone and in combination, for the prevention of childhood asthma and allergic disease. In these trials, pregnant women were randomized from mid-gestation (approximately 22–26 weeks) to receive vitamin D at 3200 IU/day in addition to the standard 400 IU/day, fish oil supplementation rich in long-chain *n* − 3 fatty acids, both interventions combined, or placebo, with supplementation continued until shortly after delivery. Offspring are being followed longitudinally for persistent wheeze, asthma, and allergic outcomes in early childhood. Although outcome data are not yet available, the study design highlights increasing interest in combined prenatal nutritional interventions targeting immune programming pathways, and supports the rationale for considering vitamin D within a broader nutritional context rather than as an isolated exposure [47].

### 4.3. Endocrine–Metabolic Roles of Vitamin D and Metabolic Programming

Beyond its classical skeletal effects, vitamin D has been increasingly investigated for its involvement in endocrine–metabolic regulation, supported by the widespread expression of the vitamin D receptor (VDR) and vitamin D–metabolizing enzymes across metabolically active tissues, including adipose tissue, liver, skeletal muscle, and pancreatic β cells [18]. Genome-wide and epigenomic analyses have demonstrated that ligand-activated VDR directly or indirectly regulates the transcription of several hundred target genes involved in cellular differentiation, lipid metabolism, insulin signaling, mitochondrial function, and inflammatory pathways [48]. Mechanistic evidence supporting a role for vitamin D in metabolic regulation derives primarily from in vitro studies and animal models focusing on adipose tissue biology and insulin-related pathways. In a comprehensive narrative review, Park et al. summarized data from cell culture experiments in preadipocyte and mature adipocyte models, as well as murine models of diet-induced obesity, showing that vitamin D signaling can influence adipocyte differentiation, lipid accumulation, inflammatory adipokine secretion, and insulin sensitivity in adipose tissue. Specifically, active vitamin D was shown to modulate key transcriptional regulators of adipogenesis, suppress pro-inflammatory signaling within adipose depots, and affect lipid storage and energy metabolism in a manner dependent on developmental stage and metabolic context [49]. Evidence from human genetic association studies further supports a potential link between vitamin D signaling and metabolic regulation. Alathari et al. reviewed studies evaluating polymorphisms in key vitamin D pathway genes, including VDR, GC, CYP2R1, and CYP27B1, and their associations with obesity-related phenotypes, insulin resistance, and type 2 diabetes risk in different populations. Overall, these data indicate that interindividual differences in vitamin D metabolism and signaling may influence metabolic traits; however, results vary considerably between studies and populations, and the available evidence does not allow causal relationships to be established [50].

Evidence from human studies during the first 1000 days derives primarily from observational pregnancy cohorts and a limited number of randomized controlled trials, with metabolic outcomes assessed mainly through growth trajectories, body composition, and adiposity-related indices in infancy and early childhood. In a large prospective cohort analysis from the Hyperglycemia and Adverse Pregnancy Outcome (HAPO) study, conducted in a North American subset of pregnant women and their term neonates, higher maternal body mass index was inversely associated with maternal late-gestation and cord-blood vitamin D concentrations, independent of glycemic status. Despite this inverse relationship, maternal and neonatal vitamin D levels were not independently associated with measures of neonatal adiposity, highlighting the strong confounding role of maternal metabolic status in studies of prenatal vitamin D and early metabolic outcomes [51]. In the multi-ethnic GUSTO prospective mother–offspring cohort from Singapore, maternal vitamin D concentrations measured during mid-pregnancy were not independently associated with birth outcomes, early postnatal growth, or adiposity during the first two years of life after adjustment for maternal BMI and other confounding factors, suggesting a limited independent contribution of prenatal vitamin D status to early metabolic outcomes [52]. Interventional evidence in infancy is provided by the Delhi Infant Vitamin D Supplementation (DIVIDS) trial, a randomized controlled study conducted in low-birthweight term infants in India, a population characterized by a high prevalence of vitamin D deficiency. Infants receiving 400 IU/day of vitamin D from birth to 6 months were followed up at 3–6 years of age; children in the supplementation group exhibited slightly lower BMI z-scores and smaller peripheral anthropometric measures, while no differences were observed in body fat percentage or functional outcomes, indicating modest and context-dependent effects on early metabolic trajectories [53]. Additional evidence comes from a Canadian randomized controlled trial by Hazell et al., conducted in healthy term infants supplemented from birth to 12 months with daily vitamin D doses ranging from 400 to 1600 IU. At 3 years of age, no significant differences were observed across dose groups in BMI or body composition assessed by DXA; however, higher achieved serum 25-hydroxyvitamin D concentrations during infancy were modestly associated with a leaner body composition, suggesting limited and context-dependent metabolic effects in vitamin D-replete populations [54]. At the level of aggregated evidence, a 2021 systematic review and meta-analysis of randomized controlled trials by Ma et al. found no consistent effects of vitamin D supplementation during pregnancy or early life on childhood growth or body composition. Modest associations were observed only in selected subgroups, particularly in settings characterized by baseline vitamin D deficiency, and substantial heterogeneity across trials was noted [55]. More recently, a 2024 systematic review and meta-analysis by Chien et al. reported that vitamin D supplementation during pregnancy was associated with modest improvements in maternal metabolic outcomes, including a reduced risk of gestational diabetes mellitus, while evidence for sustained effects on offspring metabolic or growth-related outcomes beyond birth remained limited and inconsistent [26].

### 4.4. Growth and Birth Outcomes

Fetal growth and birth outcomes are determined by a complex interplay of placental function, maternal nutritional status, and endocrine regulation. The potential role of vitamin D in these processes has been supported by mechanistic and observational evidence linking maternal vitamin D status to placental development, calcium transport, and cellular differentiation [18,20]. In a population-based prospective cohort from the Netherlands (Generation R Study), Miliku et al. examined over 7000 mother–offspring pairs with maternal 25-hydroxyvitamin D measured at a median gestational age of approximately 20 weeks. Women in the lowest quartile of 25(OH)D concentrations exhibited proportionate fetal growth restriction during late gestation and significantly higher risks of preterm birth and small-for-gestational-age (SGA) infants compared with those in the highest quartile. Notably, these associations were observed within commonly encountered ranges of vitamin D status rather than extreme deficiency, underscoring the potential relevance of suboptimal prenatal vitamin D exposure for fetal growth trajectories in general populations [56]. Consistent findings have been reported across multiple prospective cohorts and summarized in meta-analyses of observational studies. A systematic review and meta-analysis by Santamaria et al., including over 35,000 mother–offspring pairs, reported that low prenatal vitamin D status was associated with lower birth weight and a higher risk of SGA, although associations with postnatal growth parameters beyond infancy were less consistent [57]. Extending these observations, a large dose–response meta-analysis by Zhao et al., synthesizing data from more than 70 prospective studies, demonstrated a nonlinear inverse relationship between maternal 25(OH)D concentrations and the risks of preterm birth, low birth weight, and SGA. Each 25 nmol/L increase in maternal 25(OH)D was associated with a modest but significant reduction in these adverse outcomes, with the steepest risk gradients observed at concentrations below approximately 30–50 nmol/L, supporting a threshold-dependent effect during fetal growth [58]. In contrast, evidence from randomized controlled trials of prenatal vitamin D supplementation shows more variable effects on fetal growth outcomes. Large supplementation trials conducted in populations with a high prevalence of vitamin D deficiency, including those led by Roth and colleagues in South Asia, have demonstrated robust improvements in maternal and neonatal vitamin D status but only modest or inconsistent effects on birth weight, length, or SGA risk. These findings suggest that correcting deficiency alone may be insufficient to produce large shifts in anthropometric birth outcomes, particularly when other growth-limiting factors coexist [59]. Similarly, the 2019 Cochrane systematic review by Palacios et al., encompassing over 7000 pregnant women across randomized trials with heterogeneous dosing regimens and co-interventions, concluded that vitamin D supplementation during pregnancy reliably increases maternal and cord-blood 25(OH)D concentrations but has variable and generally small effects on preterm birth and low birth weight [60]. Integrating evidence across study designs, an umbrella review by Chien et al. highlighted that associations between maternal vitamin D status and adverse birth outcomes such as SGA and preterm birth are most consistently observed in observational studies, whereas interventional effects on fetal growth outcomes are modest and context-dependent. This pattern supports the interpretation that vitamin D may function as a permissive or enabling factor for optimal fetal growth—particularly under conditions of deficiency—rather than as a strong isolated determinant of birth size [26].

### 4.5. Contribution to Neurodevelopment

Vitamin D is increasingly recognized as a biologically relevant factor in early brain development through its role as a nuclear hormone regulating gene transcription and cellular differentiation within the central nervous system [18]. Evidence supporting direct responsiveness of the developing brain to vitamin D derives primarily from experimental studies conducted in animal models, in vitro neural cell systems, and analyses of human fetal brain tissue. In rodent models, expression of the vitamin D receptor (VDR) and vitamin D-metabolizing enzymes, including CYP27B1 and CYP24A1, has been demonstrated in multiple brain regions during embryonic and fetal development, including the cortex, hippocampus, and cerebellum, indicating that vitamin D signaling is active during critical phases of neurodevelopment [61]. Additional mechanistic insights derive from translational studies summarized by Ogbu et al., which integrate evidence from murine models of vitamin D deficiency or VDR dysfunction and in vitro studies of intestinal and immune cell systems. These studies show that vitamin D signaling modulates gut barrier integrity, microbiota composition, and systemic inflammatory signaling, with downstream effects on neuroimmune pathways, thereby providing an indirect biological framework linking vitamin D status to gut–brain axis-mediated influences on neurodevelopment [62]. Within the general framework of human brain development, early and mid-gestation represent periods of rapid neuronal proliferation and circuit formation [63]. Conceptual models of developmental nutrition further suggest that nutrient availability during these critical windows may influence long-term neurodevelopmental trajectories, although nutrient-specific effects vary and require empirical validation [64]. Translation of this biological plausibility to human neurodevelopmental outcomes derives predominantly from prospective observational pregnancy cohorts conducted in generally healthy term populations. In the ECLIPSES cohort, a prospective study of healthy pregnant women and their term offspring in Spain, maternal 25-hydroxyvitamin D concentrations were measured during early and late pregnancy and related to infant neurodevelopmental outcomes assessed in the first two years of life. Lower maternal 25(OH)D concentrations, particularly during the first trimester, were associated with poorer language and fine-motor development scores in infancy, whereas no consistent associations were observed with global cognitive performance, suggesting domain-specific rather than generalized neurodevelopmental effects of prenatal vitamin D exposure [65]. In a post hoc analysis of a randomized controlled trial, Rodgers et al. reported that achieving adequate maternal 25-hydroxyvitamin D concentrations during pregnancy was associated with higher language and fine-motor scores in early childhood, whereas no significant effects were observed on global cognitive outcomes, suggesting selective sensitivity of specific neurodevelopmental domains to prenatal vitamin D exposure [66]. In contrast, a double-blind randomized clinical trial conducted in healthy full-term infants in Helsinki found no differences in cognitive, language, or motor development between high-dose (1200 IU/day) and standard-dose (400 IU/day) postnatal vitamin D supplementation, indicating limited neurodevelopmental benefit of infant supplementation beyond sufficiency [67]. Preterm infants, particularly those born very or moderate-to-late preterm, represent a potentially more vulnerable subgroup, given their high prevalence of vitamin D deficiency and rapid postnatal brain maturation. A recent Italian clinical review summarized observational evidence suggesting associations between low vitamin D status and adverse neurodevelopmental features in preterm neonates, including infants born before 32–34 weeks of gestation, while emphasizing the lack of robust randomized trials and the substantial confounding from comorbidities and concurrent nutritional factors [21].

Although vitamin D supplementation consistently increases circulating 25(OH)D concentrations, the optimal dose and delivery strategy for influencing non-skeletal outcomes during pregnancy and early life remain uncertain. Variability in baseline vitamin D status, timing of supplementation, dosing regimens, and outcome measures likely contributes to the heterogeneous findings reported across randomized trials. The main characteristics of key randomized trials of vitamin D supplementation during pregnancy and early life are summarized in Table 1.

## 5. Placental and Epigenetic Mechanisms Underlying Vitamin D Programming

### 5.1. Placental Pathways

The placenta represents a critical site at which vitamin D may influence early developmental processes. In early pregnancy, components of the vitamin D system—including the vitamin D receptor (VDR) and enzymes involved in vitamin D metabolism—are expressed in placental and decidual tissues. In their comprehensive review, Ganguly et al. detailed how locally active vitamin D regulates key aspects of trophoblast biology, including proliferation, differentiation, invasion, and hormone production, processes that are essential for normal placental development and function. Specifically, the authors summarized experimental evidence derived from in vitro studies using primary human trophoblast cells, trophoblast cell lines, and ex vivo placental explant models, showing that 1,25-dihydroxyvitamin D modulates trophoblast invasion and supports appropriate placental adaptation during early gestation, suggesting a role for vitamin D in establishing a healthy maternal–fetal interface [68]. Alterations in placental vitamin D signaling have been implicated in pathological pregnancies characterized by impaired placental development. In pregnancies complicated by fetal growth restriction and preeclampsia, Murthi et al. reported reduced placental VDR expression alongside changes in markers of trophoblast turnover and placental growth. These findings suggest that disrupted placental responsiveness to vitamin D may contribute to abnormal feto-placental development in these conditions, although causality cannot be inferred from available human data [69].

At the molecular level, emerging evidence from human observational studies supports an association between maternal vitamin D status and placental gene expression. In a recent prospective cohort study, Parenti et al. examined placental tissue collected at delivery from pregnancies with well-characterized maternal vitamin D status and performed genome-wide placental transcriptomic analyses. In this study, maternal 25-hydroxyvitamin D concentrations measured during mid-pregnancy were associated with differential expression of placental genes at birth, particularly those involved in intracellular transport, transcriptional regulation, and cellular signaling pathways. Notably, these associations were observed for mid-gestation vitamin D status but not for concentrations measured at delivery, suggesting that timing of prenatal vitamin D exposure may be critical for placental molecular adaptations relevant to early developmental programming [70].

Beyond structural development and angiogenesis, vitamin D signaling also contributes to placental immune regulation. Experimental evidence derived from in vitro studies using human placental cells and ex vivo placental tissue, as well as murine models of placental inflammation, indicates that vitamin D modulates inflammatory pathways at the maternal–fetal interface, influencing innate immune responses and cytokine balance during early pregnancy. In particular, vitamin D has been shown to attenuate placental inflammatory responses to lipopolysaccharide exposure and to regulate immune-related signaling pathways, supporting the establishment and maintenance of immune tolerance within the placenta [71,72]. Vitamin D may also influence placental nutrient handling. Jia et al., using human placental tissue and trophoblast cell models, demonstrated that vitamin D stimulates the placental L-type amino acid transporter 1 (LAT1), a key mediator of essential amino acid transfer to the fetus, with relevance in preeclamptic pregnancies. These findings suggest that vitamin D signaling may directly affect placental nutrient transport capacity, thereby modulating fetal substrate availability during critical periods of growth [73]. In addition, studies of placental vitamin D metabolism indicate that the placenta actively regulates vitamin D availability within the fetoplacental unit. Using ex vivo human placental tissue, Ashley et al. showed that placental uptake and local metabolism of 25(OH)D determine the extent of vitamin D activity at the maternal–fetal interface, reinforcing the concept that the placenta functions as an autonomous site of vitamin D activation and signaling rather than a passive conduit [74].

Placental vitamin D pathways also appear to intersect with maternal metabolic conditions. Observational studies examining human placentas collected at delivery from pregnancies affected by gestational diabetes mellitus have reported altered expression of vitamin D-related genes within placental tissue, together with lower maternal and cord-blood 25(OH)D concentrations [75]. Furthermore, in an experimental animal model of preeclampsia, prenatal vitamin D_3_ supplementation was shown to reduce maternal blood pressure and to improve placental angiogenic balance, including modulation of angiogenesis-related markers, suggesting a role for vitamin D in placental vascular development under pathological conditions [76,77]. In a separate murine experimental model, placenta-specific overexpression of CYP11A1 resulted in autism-like behavioral phenotypes in offspring, accompanied by altered placental steroid hormone biosynthesis along a proposed placenta–brain axis; importantly, vitamin D intervention in this animal model rescued both placental molecular alterations and offspring neurobehavioral outcomes, indicating that placental endocrine pathways may represent a target of vitamin D-dependent modulation during development [78].

### 5.2. Epigenetic Mechanisms

Epigenetic regulation represents a key mechanism through which early-life vitamin D exposure may exert long-lasting effects on developmental trajectories. Vitamin D acts via the vitamin D receptor (VDR), a ligand-activated transcription factor that binds vitamin D response elements and recruits chromatin-modifying complexes, thereby influencing gene expression beyond direct transcriptional control. As reviewed by Snegarova et al., vitamin D signaling can modulate both DNA methylation and histone-related processes, shaping chromatin accessibility in a context- and time-dependent manner [79]. During periods of rapid cellular differentiation, such as fetal life and early infancy, vitamin D-dependent epigenetic remodeling appears particularly relevant. Carlberg and colleagues highlighted that vitamin D signaling influences DNA methylation at specific CpG sites in genes involved in immune, metabolic, and developmental pathways, supporting a role for vitamin D in establishing stable epigenetic marks during early development [48]. Consistent with this framework, Ong et al. reported associations between vitamin D status and DNA methylation patterns across the life course, reinforcing the concept of vitamin D as a modulator of epigenetic programming [80]. Human interventional data provide direct support for these mechanisms. In a post hoc analysis of a randomized controlled trial, Chen et al. demonstrated that maternal vitamin D_3_ supplementation during pregnancy was associated with reduced epigenetic gestational age acceleration at birth, suggesting a favorable influence on fetal epigenetic aging and intrauterine developmental timing [81]. The placenta represents a key tissue in which vitamin D-related epigenetic mechanisms may operate. Placental and cord-blood DNA methylation profiles have been shown to reflect maternal nutritional and metabolic exposures, with potential implications for fetal development. In this context, altered placental DNA methylation patterns observed in metabolically complicated pregnancies further support the sensitivity of the placental epigenome to early environmental cues, including vitamin D-related pathways [82]. Experimental evidence also suggests that vitamin D can modulate histone-dependent inflammatory and stress-response pathways, providing mechanistic insight into its potential neuroprotective and immunomodulatory roles during development [83]. Finally, emerging nutritional epigenomics research supports the broader concept that maternal nutrition during pregnancy and lactation—including vitamin D status—can shape the infant epigenome with downstream effects on immune development and disease susceptibility. As reviewed by Di Costanzo et al., epigenetic modifications induced by early nutritional exposures may influence immune tolerance and allergic risk, reinforcing the relevance of vitamin D as part of an integrated nutritional programming framework [84]. While these experimental and translational findings provide important biological plausibility for vitamin D-mediated developmental programming, direct evidence linking these molecular mechanisms to long-term clinical outcomes in humans remains limited. Most available data derive from experimental models or observational studies, highlighting the need for integrative research combining mechanistic insights with longitudinal human cohorts and randomized trials.

## 6. Vitamin D Status in Intra-Uterine Life, Neonatal Life and Early Childhood

### 6.1. Definitions and Thresholds of Vitamin D Status

When interpreting vitamin D status during the first 1000 days of life, particular caution is required, as most commonly used thresholds were not derived from pregnancy, neonatal, or infant populations. Vitamin D status is consistently defined across international guidelines and consensus documents by serum concentrations of 25-hydroxyvitamin D [25(OH)D], which is recognized as the best biomarker reflecting vitamin D intake, cutaneous synthesis, and body stores. However, substantial heterogeneity persists in the proposed cut-off values used to define deficiency, insufficiency, and sufficiency, largely due to differences in target outcomes (skeletal vs. non-skeletal), population-based versus clinical approaches, and the limited availability of randomized controlled trials linking specific 25(OH)D thresholds to hard health outcomes [85,86]. Historically, the Institute of Medicine (IOM/NAM) proposed a serum 25(OH)D concentration of ≥20 ng/mL (50 nmol/L) as sufficient to meet the needs of nearly all individuals for bone health, with values below 20 ng/mL classified as insufficient and concentrations <12 ng/mL indicative of deficiency [87]. These thresholds were derived primarily from adult populations and from endpoints related to calcium absorption and prevention of osteomalacia and rickets [87,88]. In the general population, the UK Scientific Advisory Committee on Nutrition (SACN) defines vitamin D deficiency as serum 25(OH)D concentrations below 25 nmol/L (10 ng/mL), identifying this threshold as the level at which the risk of adverse musculoskeletal outcomes increases at a population level [85]. In contrast, the European Calcified Tissue Society (ECTS) position statement classifies vitamin D deficiency as serum 25(OH)D <50 nmol/L (20 ng/mL) and severe deficiency as <30 nmol/L (12 ng/mL), considering concentrations ≥50 nmol/L generally sufficient to maintain bone health in adults [89]. Notably, the most recent 2024 Endocrine Society clinical practice guideline has moved away from endorsing fixed 25(OH)D cut-offs for the general healthy population. The guideline concludes that current evidence does not support a single serum level consistently associated with clinically meaningful benefits for disease prevention and discourages routine 25(OH)D testing in healthy adults younger than 75 years [90]. This paradigm shift is further supported by a 2024 international consensus, which emphasizes that commonly used categories of deficiency (<50 nmol/L), insufficiency (50–75 nmol/L), and sufficiency (>75 nmol/L) are largely pragmatic constructs rather than evidence-based thresholds, and should therefore be interpreted cautiously and contextually [86].

In pediatric populations, vitamin D status thresholds are primarily derived from evidence related to the prevention of nutritional rickets and impaired skeletal mineralization, particularly during the critical window of the first 1000 days of life. The Global Consensus Recommendations on the Prevention and Management of Nutritional Rickets define vitamin D sufficiency as serum 25(OH)D >50 nmol/L (20 ng/mL), insufficiency as 30–50 nmol/L (12–20 ng/mL), and deficiency as <30 nmol/L (12 ng/mL), thresholds that are explicitly linked to the risk of rickets in infants and children [28]. In this context, Corsello et al. (2023) highlight that vitamin D status in early life should not be interpreted as a direct extension of adult-derived models, emphasizing that serum 25(OH)D concentrations in infants and young children must be contextualized within developmental stage, prenatal exposure, postnatal nutrition, and calcium intake, particularly during periods of rapid growth [91]. Recent pediatric guidelines and narrative reviews consistently report that maternal serum 25-hydroxyvitamin D [25(OH)D] concentrations during pregnancy are a major determinant of neonatal and infant vitamin D status, because cord blood and early postnatal 25(OH)D levels closely reflect maternal circulating concentrations at delivery. In particular, Pludowski et al. summarize observational cohort data and intervention studies showing that maternal 25(OH)D concentrations below 50 nmol/L are associated with a high prevalence of neonatal deficiency and reduced infant vitamin D stores during the first months of life. The authors further state that higher-risk pediatric groups—including preterm infants, children with chronic inflammatory or malabsorptive diseases, and those with limited sunlight exposure—may require individualized interpretation of serum 25(OH)D concentrations rather than fixed cut-offs [92]. This position is consistent with the clinical review by Cediel et al. (2018), which underscores that in pediatric practice serum 25(OH)D values should always be interpreted in conjunction with clinical signs, dietary calcium intake, growth velocity, and radiological or biochemical markers of bone metabolism, rather than used as isolated diagnostic thresholds [93].

### 6.2. Prevalence of Vitamin D Deficiency

Vitamin D deficiency is widely prevalent across pregnancy, neonatal life, and early childhood, although estimates vary substantially due to differences in latitude, ethnicity, lifestyle, and the cut-offs applied [94]. A recent pooled analysis of 7.9 million participants from population-based studies conducted between 2000 and 2022 reported a global prevalence of vitamin D deficiency (<20 ng/mL) of approximately 28%, with the highest rates observed in South Asia and the Middle East, where deficiency frequently exceeds 50–60%. Importantly, women of reproductive age, pregnant women, and young children were consistently identified as among the subgroups at greatest risk, regardless of geographic region [95]. Consistent with these findings, a meta-analysis including over 54,000 pregnant women found that 54% had serum 25(OH)D levels below 20 ng/mL, with particularly high prevalence documented in regions such as South Asia, the Middle East, and parts of Africa [96]. In Europe, vitamin D deficiency remains common despite public health recommendations. Approximately 18% of the population has serum 25(OH)D concentrations below 30 nmol/L [95], a finding consistent with recent data from the UK Biobank, where 13% of participants were classified as severely deficient, with 25(OH)D levels ranging from 10 to 25 nmol/L [97]. High prevalence rates have been documented among women, especially during pregnancy [98,99] and lactation [100]. Moreover, recent pediatric studies indicate that vitamin D deficiency is highly prevalent not only at birth but throughout early childhood. In a 2024 cohort study, a high prevalence of deficiency was observed among both mothers and their neonates, with neonatal 25(OH)D concentrations frequently below sufficiency thresholds, highlighting a strong correlation between maternal and newborn status, yet also suggesting independent neonatal insufficiency [101]. Consistent with this, Delair et al. reported a high prevalence of vitamin D insufficiency and deficiency in cord blood samples, with marked variability according to maternal status and season of birth [102]. Seasonal and ethnic factors further influence prevalence, with up to 70–85% of neonates having 25(OH)D <50 nmol/L in some populations, particularly in winter-born infants [103]. Among infants and children, recent data show that vitamin D deficiency remains a concern, with up to 65% of infants in a 2025 Kazakhstan cohort classified as deficient, and notable prevalence gradients across age groups, such as 13.7% in preschoolers, 18.2% in school-aged children, and 23.9% in adolescents [104,105]. Large multicenter data from China also indicate that deficiency increases with age, from approximately 3.7% in the youngest children to 43.5% in older adolescents [54].

### 6.3. Determinants of Early-Life Vitamin D Status

Early-life vitamin D status reflects a dynamic interplay between maternal stores during pregnancy and postnatal exposures across the first 1000 days. During gestation, maternal sunlight exposure and season remain major drivers of circulating 25(OH)D concentrations, thereby influencing fetal supply, as placental transfer results in cord-blood levels that closely reflect maternal concentrations [106]. Maternal adiposity represents another consistent determinant: observational studies have demonstrated progressively lower maternal and cord-blood 25(OH)D concentrations with increasing maternal BMI, supporting volumetric dilution and adipose sequestration as mechanisms that may limit fetal bioavailability [51]. From a nutritional programming perspective, maternal vitamin D intake through diet and supplementation constitutes the most readily modifiable prenatal determinant. Randomized controlled trials conducted in vitamin D-deficient populations demonstrate that higher-dose antenatal cholecalciferol supplementation significantly increases maternal 25(OH)D concentrations in late pregnancy and raises cord-blood levels at birth, effectively modifying the newborn’s initial vitamin D status [107]. Consistently, the multicentre MAVIDOS trial showed that pregnancy supplementation modifies maternal vitamin D status and downstream neonatal outcomes, reinforcing the concept that intrauterine vitamin D exposure can be modified during critical developmental windows [24].

After birth, determinants of vitamin D status shift toward infant feeding practices and adherence to supplementation guidance. Breast milk typically provides limited vitamin D unless maternal intake is high; indeed, it is well established that exclusively breastfed infants are at increased risk of vitamin D deficiency, which has been associated with hypocalcemia and nutritional rickets, particularly in the absence of adequate vitamin D supplementation [108]. Accordingly, infant vitamin D status is strongly influenced by whether the infant receives direct supplementation or whether maternal high-dose supplementation during lactation is used as an alternative strategy [30]. In this context, a randomized controlled trial by Ruangkit et al. in exclusively breastfed infants documented a high prevalence of suboptimal 25(OH)D concentrations at around 6 months in the absence of supplementation, underscoring feeding mode as a key postnatal determinant [109]. Emerging evidence further suggests that maternal dietary patterns during lactation, including intake of vitamin D-rich foods and fortified products, contribute modestly to infant status but are insufficient alone to meet infant requirements without supplementation [30,110]. As complementary feeding is introduced, dietary vitamin D intake may increase; however, several observational studies indicate that complementary foods often provide inadequate amounts of vitamin D, particularly in settings where fortified foods are limited or dietary diversity is low. Consequently, supplementation frequently remains necessary beyond the exclusive breastfeeding period to maintain adequate 25(OH)D concentrations during infancy and early childhood [111,112].

Beyond feeding practices, infant and toddler vitamin D status is further shaped by seasonality and outdoor exposure, which interact with latitude, urban living, and cultural practices that limit UVB exposure, leading to predictable risk gradients across populations [113]. Finally, environmental and genetic factors further modulate susceptibility. Variants in vitamin D pathway genes—particularly those affecting vitamin D-binding protein (GC/DBP) and enzymes involved in vitamin D activation—contribute to inter-individual differences in circulating 25(OH)D. Importantly, in pediatric populations, a randomized controlled trial by Cerullo et al. demonstrated that infant DBP genotype influences both 25(OH)D concentrations and the biochemical response to high-dose vitamin D supplementation [114]. Maternal–neonatal DBP polymorphism profiles have also been investigated as contributors to variability in maternal and neonatal vitamin D status [115]. Together, these determinants help explain why the first 1000 days represent a uniquely modifiable window during which optimizing vitamin D exposure may influence early developmental trajectories.

## 7. Current Recommendations and Guidelines

Current recommendations for vitamin D intake across pregnancy, lactation, and early infancy aim primarily to prevent nutritional rickets and support normal skeletal development, while acknowledging that evidence for non-skeletal prevention outcomes is less consistent and often context-dependent [90,116]. Because different organizations have proposed varying thresholds for vitamin D sufficiency, the interpretation of study findings may depend on the cut-off values applied. In this review, references to deficiency or sufficiency reflect the thresholds used within the original studies, which often align with guideline frameworks such as those proposed by the IOM, SACN, or ECTS [85,86,87,89,90].

### 7.1. Pregnancy

The 2024 Endocrine Society Clinical Practice Guideline suggests empiric vitamin D supplementation during pregnancy because of potential benefits on pregnancy and neonatal outcomes (e.g., preeclampsia, preterm birth, SGA, neonatal mortality). In the trials informing this recommendation, vitamin D doses ranged from 600 to 5000 IU/day (daily equivalent), with an estimated weighted average of ~2500 IU/day. The same guideline suggests against routine 25(OH)D testing during pregnancy in generally healthy pregnant individuals, noting that outcome-specific thresholds have not been established [90].

### 7.2. Neonates and Infants

International pediatric prevention strategies consistently recommend routine infant supplementation, especially for breastfed infants, because human milk typically contains insufficient vitamin D unless maternal intake is very high [28]. The 2022 AAP breastfeeding policy statement reiterates 400 IU/day for infants, and notes maternal 6400 IU/day as an alternative strategy to direct infant supplementation in breastfeeding dyads [117]. The Global Consensus Recommendations on Nutritional Rickets indicate that 400 IU/day during infancy is sufficient to prevent radiographic signs of rickets in the short term (up to 12 months), including among infants born with low vitamin D status [28]. This recommendation is consistently endorsed at the international level by the World Health Organization Department of Nutrition for Health and Development [118], ESPGHAN [119], the European Academy of Paediatrics [120], as well as regional guidelines from the United Arab Emirates [121] and Central Europe [122]. Across these guidelines, a daily supplementation of 400 IU is uniformly considered both safe and effective for the prevention of nutritional rickets and for maintaining adequate vitamin D status during infancy. Furthermore, the Italian pediatric consensus, developed by the Italian Pediatric Society and related professional bodies, such as the Società Italiana di Pediatria Preventiva e Sociale (SIPPS) and the Federazione Italiana Medici Pediatri (FIMP), recommends routine vitamin D supplementation for all newborns regardless of feeding modality, starting from birth and continuing throughout the first year of life. According to this consensus, term infants without specific risk factors for vitamin D deficiency should receive 400 IU/day, whereas higher doses—up to 1000 IU/day—may be considered in the presence of recognized risk factors for deficiency [4,123,124]. Preterm infants represent a distinct population with specific requirements due to reduced transplacental vitamin D transfer and limited body stores at birth. Specific recommendations apply to preterm infants, who represent a population at increased risk of vitamin D deficiency due to reduced transplacental transfer and higher nutritional requirements [21]. For preterm neonates with a birth weight below 1500 g, a total daily vitamin D intake of 200–400 IU is generally recommended, accounting for all sources, including parenteral nutrition, fortified human milk, and preterm formula. Once very-low-birth-weight infants reach a body weight of at least 1500 g and achieve full enteral feeding, as well as in preterm infants born with a birth weight ≥ 1500 g, vitamin D supplementation in the range of 400–800 IU/day is advised. After reaching a postconceptional age of 40 weeks, vitamin D supplementation strategies align with those recommended for healthy term infants [4].

### 7.3. Toddlers (12–24 Months) and Childhood

Current pediatric consensus statements from the European Society for Paediatric Gastroenterology, Hepatology and Nutrition (ESPGHAN), the European Academy of Paediatrics, and the Global Consensus on Nutritional Rickets indicate that, from 12 months of age onward, routine vitamin D supplementation should be individualized and primarily targeted to children with recognized risk factors for deficiency, including limited sun exposure, darker skin pigmentation, chronic diseases affecting absorption or metabolism, obesity, and use of medications that interfere with vitamin D metabolism (e.g., antiepileptics or glucocorticoids) [4,28,64,119,120]. For otherwise healthy toddlers (12–24 months) and young children without specific risk factors, vitamin D supplementation may be limited to periods of reduced ultraviolet B exposure, typically during autumn and winter months, at doses of 400 IU/day, which are considered sufficient to maintain adequate vitamin D status in most settings [4,119,120]. In contrast, continuous year-round supplementation is recommended for children from toddler age through early childhood who have persistent risk factors for deficiency, generally at daily doses ranging from 400 to 600 IU, depending on age, baseline vitamin D status, and environmental factors [4,28]. Higher vitamin D intakes, commonly in the range of 800–1000 IU/day, are advised for selected pediatric populations, including children receiving medications that accelerate vitamin D catabolism of those with chronic malabsorption, in line with ESPGHAN, Endocrine Society, and Italian pediatric consensus recommendations [4,88,119,123,124].

## 8. Knowledge Gaps and Future Directions

Despite the growing body of literature on vitamin D across pregnancy, neonatal life, and early childhood, several important knowledge gaps remain, limiting the translation of existing evidence into precise, outcome-oriented recommendations. A central unresolved issue concerns the lack of standardized definitions of vitamin D sufficiency that are biologically meaningful across different tissues, developmental stages, and health outcomes. Current thresholds are largely derived from skeletal endpoints, while emerging data suggest that extraskeletal and programming-related effects may operate within different concentration ranges and exhibit tissue- and timing-specific sensitivity [90,95].

Another major limitation relates to the heterogeneity of available evidence. Observational studies consistently report associations between early-life vitamin D status and outcomes spanning growth, immune function, metabolic regulation, and neurodevelopment; however, causality remains difficult to establish due to residual confounding, co-exposures, and strong correlations with socioeconomic status, maternal diet, sun exposure, BMI and other lifestyle factors [45,57]. Conversely, randomized controlled trials—while essential for causal inference—often vary substantially in baseline vitamin D status, dosing regimens, timing of intervention, and outcome selection, resulting in mixed and sometimes inconclusive findings [25,45]. This discrepancy underscores the need for trials specifically designed to test developmental programming hypotheses rather than short-term deficiency correction.

Timing of exposure represents a further critical gap. Accumulating evidence suggests that vitamin D effects may be highly dependent on specific developmental windows, particularly early to mid-pregnancy and the neonatal period [118,120]. However, few studies have systematically compared the impact of supplementation initiated at different gestational stages or distinguished prenatal from postnatal effects, limiting our ability to define optimal intervention windows.

Mechanistic understanding, while advancing, also remains incomplete. Experimental and translational studies implicate placental vitamin D signaling, epigenetic modifications, immune modulation, and endocrine–metabolic pathways as plausible mediators of long-term programming effects [71,81,82]. Yet direct links between these molecular changes and clinically relevant long-term outcomes in humans are still scarce. Integrative approaches combining epigenetic, transcriptomic, metabolomic, and microbiome data with longitudinal phenotyping may help bridge this gap.

Population heterogeneity further complicates interpretation. Vitamin D status and the prevalence of deficiency vary widely across populations due to differences in latitude, sun exposure, dietary intake, ethnicity, and national supplementation policies. Consequently, results observed in specific geographic or demographic settings may not be directly applicable to other populations with different environmental and nutritional contexts [107,111,115]. Future research should prioritize stratified analyses and precision-nutrition approaches to identify subgroups most likely to benefit from targeted supplementation strategies.

Finally, long-term follow-up data remain limited. Many trials assess outcomes in infancy or early childhood, whereas the developmental programming framework predicts effects that may only become apparent later in childhood, adolescence, or adulthood. Well-designed prospective cohorts and extended follow-up of existing randomized trials are therefore essential to clarify whether early-life vitamin D exposure meaningfully alters lifelong health trajectories. Although many studies assessing vitamin D exposure during the first 1000 days of life report outcomes measured in infancy or early childhood, these early phenotypes are often considered intermediate indicators within the developmental origins framework [125,126]. Such early-life outcomes may reflect biological processes that influence long-term health trajectories, although direct evidence linking early vitamin D status to adult disease risk remains limited. Table 2 summarizes the available evidence across outcome domains and developmental windows, highlighting differences in consistency and strength of evidence between skeletal and non-skeletal outcomes, and emphasizing the context-dependent nature of reported associations. The categories “strong,” “moderate,” and “limited” evidence were assigned based on a qualitative assessment of the consistency of findings across studies and the methodological design of the available evidence (e.g., randomized trials versus observational studies), with greater weight given to higher-quality study designs, rather than on predefined quantitative thresholds.

In summary, available evidence suggests that future research may benefit from moving beyond uniform thresholds toward approaches that consider specific outcomes, developmental timing, and population characteristics. Addressing these aspects could help refine existing recommendations and improve understanding of the potential role of vitamin D in developmental programming during the first 1000 days of life.

## 9. Conclusions

Vitamin D is essential for skeletal development during the first 1000 days of life, and its role in preventing deficiency-related skeletal disorders is well established and supported by consistent clinical and epidemiological evidence. Accordingly, current clinical recommendations for infants include routine vitamin D oral supplementation, typically at a dose of around 400 IU/day, primarily to ensure adequate skeletal health and prevent deficiency-related disorders such as rickets.

In contrast, potential effects on immune function, metabolic regulation, and neurodevelopment remain an area of active investigation, supported by emerging observational and mechanistic data, although findings from randomized trials remain heterogeneous. Evidence from observational and interventional studies suggests that the benefits of supplementation are most evident in populations with baseline vitamin D deficiency or increased vulnerability.

Within the developmental origins framework, vitamin D fulfills several biological plausibility criteria as a potential early-life programming factor. However, current human evidence is not yet sufficient to establish a definitive causal role for most non-skeletal outcomes. Future research specifically designed to address timing of exposure, dose–response relationships, residual confounding, and long-term follow-up will be essential to clarify whether optimizing vitamin D status during the first 1000 days of life can meaningfully modify lifelong health trajectories.

## Figures and Tables

**Table 1:** Key randomized trials of vitamin D supplementation during pregnancy and early life.

Study	Population	Timing of Supplementation	Dose	Baseline Status	Main Outcome
Cooper et al. (MAVIDOS) [24]	Pregnant women	Mid-pregnancy to delivery	1000 IU/day	Mixed vitamin D status; seasonal effect	Higher neonatal bone mineral content
Weiss et al. (VDAART) [45]	Pregnant women at high allergy risk	Early pregnancy	4400 IU/day	Mixed baseline status	No clear reduction in asthma overall
Grant et al. [44]	Mother–infant pairs	Pregnancy + Infancy	1000–2000 IU/day pregnancy + 400–800 IU/day infancy	Mixed population	Lower sensitization to aeroallergens
DIVIDS trial [53]	Low-birth-weight infants	Birth to 6 months	400 IU/day	High deficiency prevalence	Modest effects on BMI trajectories
Hazell et al. [54]	Healthy term infants	Birth to 12 months	400 vs. 1600 IU/day	Vitamin D-replete population	No significant differences in body composition
Tuovinel et al. [67]	Healthy term infants	Infancy	400 vs. 1200 IU/day	Generally sufficient population	No neurodevelopmental differences

**Table 2:** Summary of evidence on vitamin D exposure and outcome domains across the first 1000 days of life.

Developmental Window	Outcome Domain	Key Findings	Consistency of Evidence	Key References
Pregnancy/Birth	Growth and birth outcomes	Lower maternal 25(OH)D associated with lower birth weight and SGA; dose–response associations in observational studies; inconsistent effects in pregnancy RCTs	Moderate (observational > RCTs)	[5,26,52,56,58,59,107]
Pregnancy/Neonatal/Infancy	Skeletal development and bone health	Severe vitamin D deficiency associated with nutritional rickets; infant supplementation improves 25(OH)D and Ca/P balance; context-dependent effects on BMC/BMD in pregnancy RCTs	Strong (rickets prevention); Moderate (BMC/BMD)	[4,24,25,26,27,28,29,30,31,32]
Neonatal/Infancy/Early childhood	Immune-related outcomes	Vitamin D supplementation associated with reduced ARTI risk; greater effects observed with daily dosing and deficiency; inconsistent findings for allergy and asthma outcomes	Moderate (regimen- and baseline-dependent)	[41,42,43,44,45,46]
Infancy/Toddlerhood	Endocrine-metabolic outcomes	Associations reported with growth trajectories and adiposity; maternal BMI identified as major confounder; no consistent metabolic effects shown in RCT meta-analyses	Limited-moderate (heterogeneous endpoints)	[51,52,53,54,55]
Pregnancy/Infancy	Neurodevelopment	Selective domain associations reported in pregnancy cohorts; limited effects reported in infant supplementation RCTs; evidence in preterm infants remains limited	Limited	[21,65,66,67]

Abbreviations: 25(OH)D, 25-hydroxyvitamin D; SGA, small for gestational age; RCT, randomized controlled trial; BMC, bone mineral content; BMD, bone mineral density; ARTI, acute respiratory tract infection; BMI, body mass index.

## Data Availability

No new data were created or analyzed in this study.
